# Supplementary material for: Low-dose decitabine priming endows CAR T cells with enhanced and persistent antitumour potential via epigenetic reprogramming
Source: Nat Commun. 2021 Jan 18;12:409. doi: 10.1038/s41467-020-20696-x (PMC7814040; doi:10.1038/s41467-020-20696-x)
Supplement: Supplementary file 1 — Supplementary Information [file 41467_2020_20696_MOESM1_ESM.pdf]

Supplementary Figures

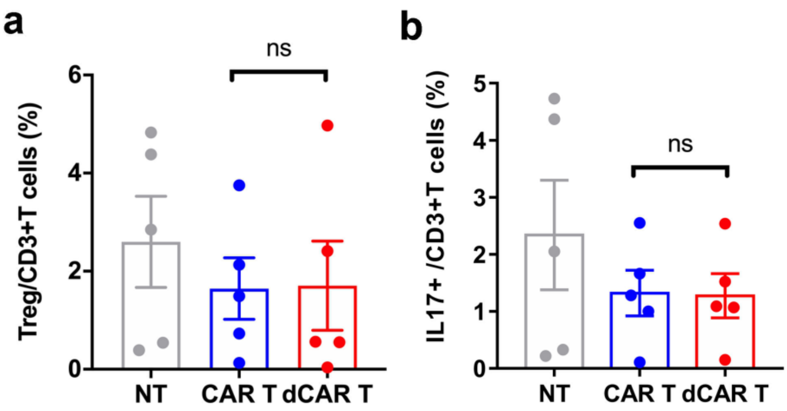

Supplementary Figure 1. The populations of regulatory T cells (Tregs) and Th17 cells does not differ between the dCAR T and CAR T cells. a, Histogram plots of Tregs frequency on CAR T and dCAR T cells after 10 days of cell culture. CD4+CD25+Foxp3+ cells were defined as Tregs in here. **b,** Histogram plots of IL17-positive CAR T and dCAR T cells after 10 days of cell culture. All data are shown as the mean ± s.e.m. from 5 donor-derived T cell samples in 3 independent experiments. All P values were calculated using two-tailed paired t-tests, ns, no significant.

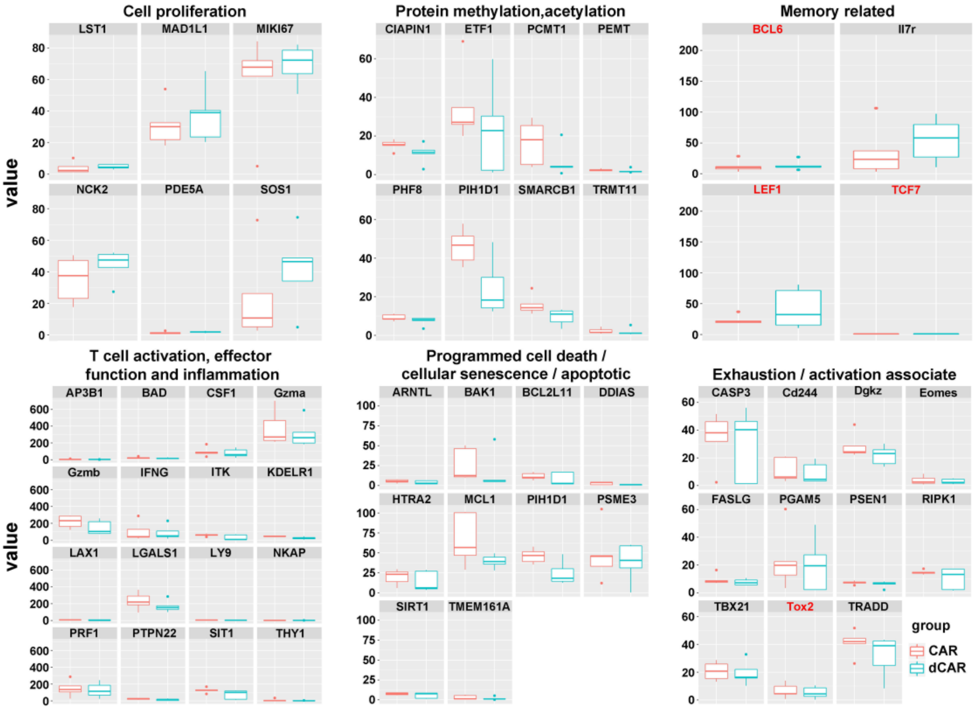

Supplementary Figure 2. Differential gene expression between dCAR T cells and CAR T cells. All samples were collected on day 10 of cell culture. Box map of differentially gene values among dCAR T cells compared to CAR T cells, median ± interquartile range of gene value; whiskers, 1.5× interquartile range. The upper whisker extends from the hinge to the largest value no further than 1.5 × IQR from the hinge (where IQR is the inter-quartile range, or distance between the first and third quartiles). The lower whisker extends from the hinge to the smallest value at most 1.5 × IQR of the hinge. Data beyond the end of the whiskers are called "outlying" points and are plotted individually. Pairwise comparisons were performed using a two-tailed paired t-tests. (Red color represents TF), (n = 5 replicates per group). Exact P values are available in the Source Data File. Source data are provided as a Source Data file.

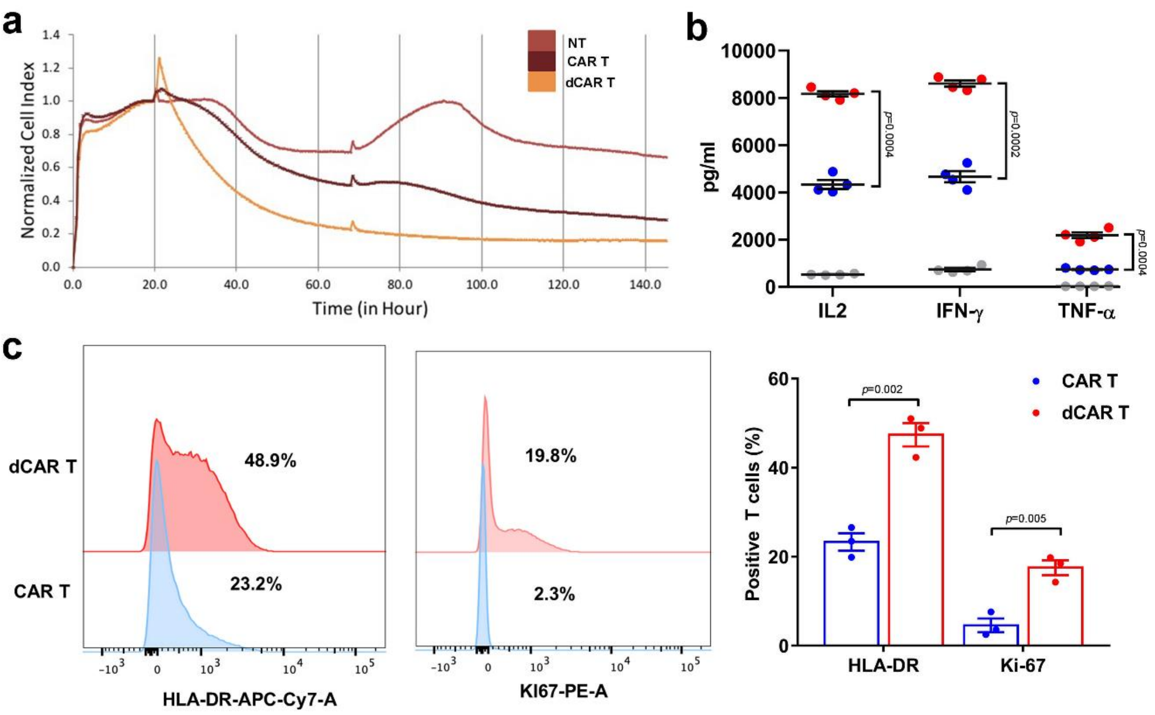

Supplementary Figure 3. Tandem dCAR T cells (target CD19 and CD20) exhibit enhanced anti-tumour capacities in vitro. a, Continuous graphical output of cell index values determined using the xCELLigence Impedance system from NT, tandem CAR T and tandem dCAR T cells co-cultured with Raji cells at an effector-to-target (E:T) ratio of 1:5 up to the 140-h time point. **b,** Cytokine production by NT, tandem CAR T and d-tandem CAR T cells (treated with DAC) co-cultured with Raji cells at an E:T ratio of 1:1 for 24 hours measured by ELISA (n = 4). Data are presented as the mean ± s.e.m.. Two-tailed, unpaired t-test was used for statistical analysis. **c,** Representative histograms: The HLA-DR and Ki67 expression of tandem CAR T cells coculture with Raji cells after 24 hours. Bar graph of the percentage of the HLA-DR and Ki67 expression of tandem CAR T cells after coculture with Raji cells at an E:T ratio of 1:1 for 24 hours (n=3). Data are means ± s.e.m. Two-tailed, paired t-test was used for statistical analysis.

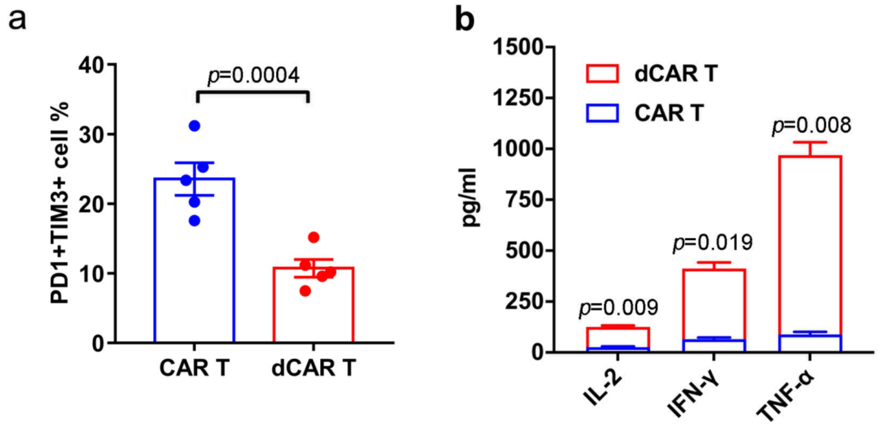

Supplementary Figure 4. dCAR T cells stimulated by antigen for long term maintain cell biology function. CAR T and dCAR T cells were continuous co-cultured with Raji cells at an E:T ratio of 5:1 for two weeks, then CAR T and dCAR T cells were collected and re-co-cultured with Raji cells at E:T=1:1 for 24 hours. **a**, The CD3+TIM3+PD1+ expression of CAR T and dCAR T cells was evaluated after 24 hours of coculture with Raji cells at an E:T ratio of 1:1. Data are shown as the mean ± s.e.m. from 5 donor-derived T cell samples in 3 independent experiments. **b,** Cytokine production was measured by Luminex assays according to the manufacturer's instructions (n=3). Data are shown as the mean ± s.e.m.. P values for all panels were calculated by two-tailed, paired t-tests.

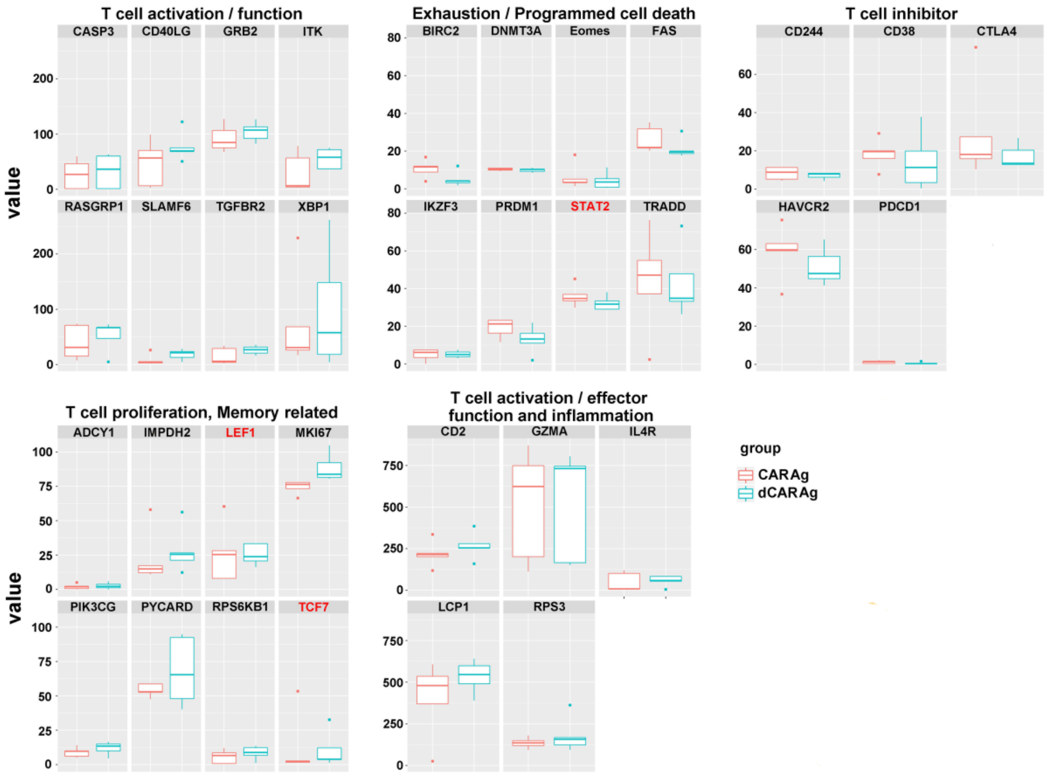

Supplementary Figure 5. Differential gene expression between dCAR T cells and CAR T cells after antigen stimulation. All samples were collected 24 hours after co-culture with Raji cells at an E:T ratio of 1:1. Box means differentially gene values among dCAR T cells compared to CAR T cells, median ± interquartile range of gene value; whiskers, 1.5× interquartile range. The upper whisker extends from the hinge to the largest value no further than 1.5×IQR from the hinge. The lower whisker extends from the hinge to the smallest value at most 1.5× IQR of the hinge. Data beyond the end of the whiskers are called "outlying" points and are plotted individually. Pairwise comparisons were performed using a two-tailed, paired, t-tests. (Red color represents TF). (n = 5 replicates per group). Exact p values are available in the Source Data File. Source data are provided as a Source Data file.

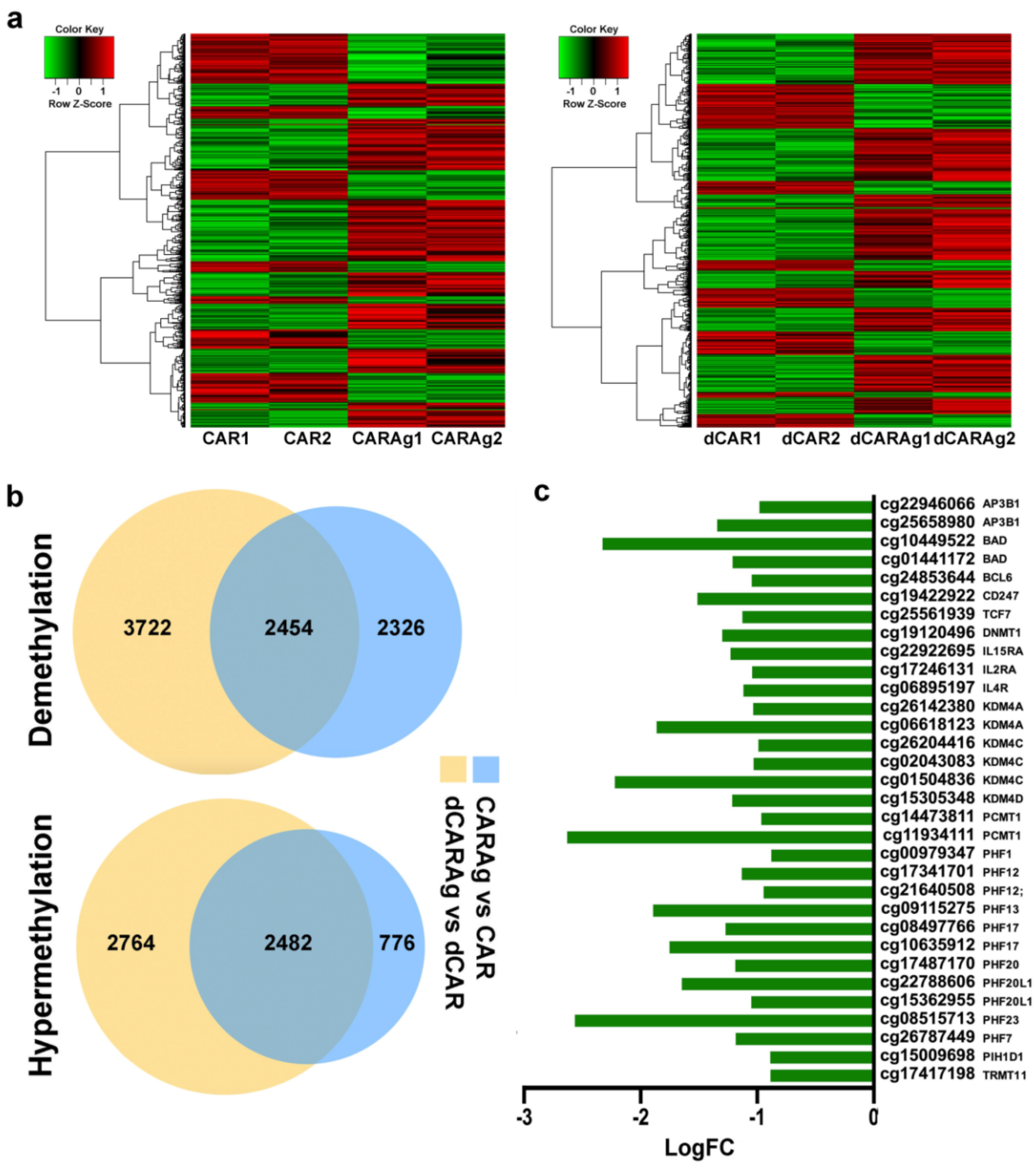

Supplementary Figure 6. The unique reprogramming effect on CAR T cells after antigen stimulation. a, Hierarchical clustering analysis of significant differentially methylated CpG sites before and after antigen stimulation of CAR T cells (**Left panel**) and dCAR T cells (**Right panel**). Hypermethylated CpG sites are shown in red; hypomethylated CpG sites are shown in green. **b,** Venn diagram representing the overlap of the different promotor-associated CpG sites in the two groups of CAR T cells before and after persistent antigen stimulation. **c,** The data show the differences and consistency with the regulation of the transcriptome in promotor-associated CpG sites in the dCAR T group before and after antigen stimulation, which did not overlap with the CAR T group. All significantly differentially CpG sites were calculated by Generalized Linear Models (v3.36.2) (P value < 0.05, fold change (log2 scale) ≥ 1 or ≤ -1).

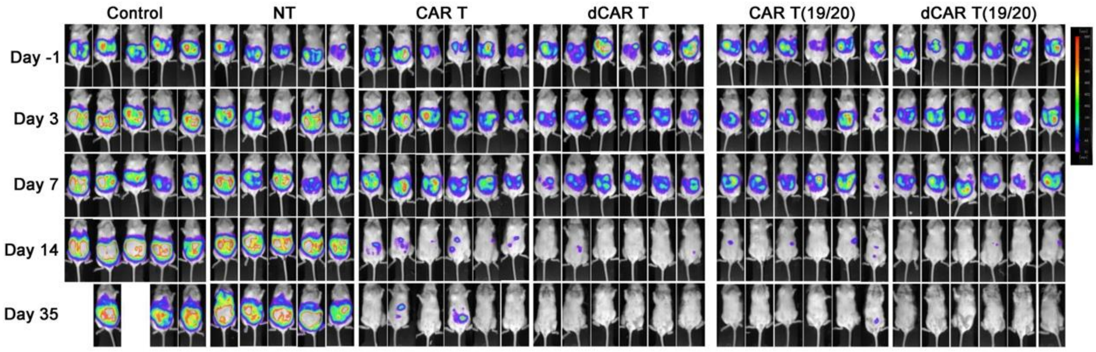

Supplementary Figure 7. dCAR T cells exhibit enhanced anti-tumour responses in normal treatment cell dose. Related to Figure 6. A total of 1×10^6 luciferase-expressing Raji cells mixed with Matrigel were intraperitoneally (i.p.) transferred to NPG mice. Tumour growth was monitored by bioluminescence (BLI). After engraftment was confirmed by increased photon activity after 7 days, 10^7 effector cells (or the same volume normal saline as a control) were injected (i.v), after which BLI showing the tumour burden was monitored with the schedule shown. BLIs of mouse tumour burdens at the indicated time points are representative of all experiments (n = 6 mice per group, the results were pooled from two independent experiments). CAR T (19/20): tandem CAR T. Control: the same volume normal saline.

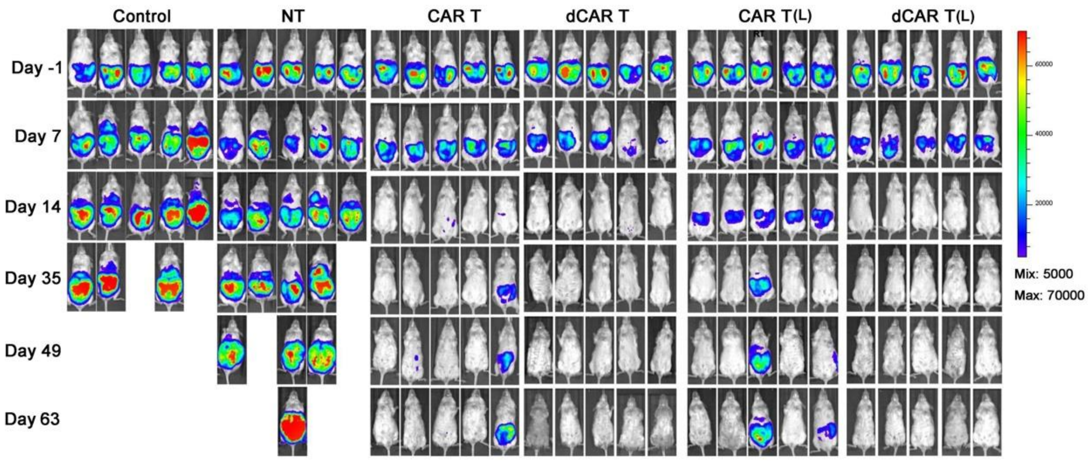

Supplementary Figure 8. dCAR T cells display enhanced in vivo antitumour activity against a large tumour burden. Related to Figure 7. A total of 1×10^6 luciferase-expressing Raji cells mixed with Matrigel were intraperitoneally (i.p.) transferred to NPG mice. Tumour growth was monitored by BLI. After 21 days, effector cells (or the same volume normal saline as a control) were injected (i.v.), after which the tumour burden was assessed by monitoring BLI with the schedule shown. Tumour burden in mice treated with 1×10^7 CD19-CAR T or dCAR T cells and 1×10^6 CD19-CAR T or dCAR T cells (L). BLIs of mouse tumour burdens at the indicated time points are representative of all experiments (n = 5 mice per group). Control: the same volume normal saline.

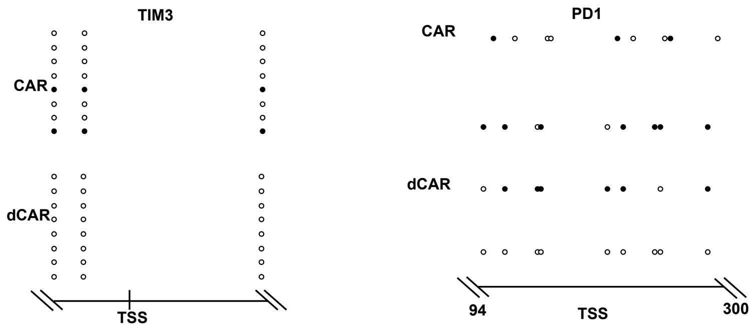

Supplementary Figure 9. Bisulfite sequencing of *PD1* and *TIM3* genes in CAR T cells and dCAR T cells. DNA from CAR T and dCAR T cells cultured for 10 days were sequenced by sodium bisulfite treatment. Representative graph: The double-headed arrow indicates the region of the methylation-specific PCR (MSP) product. Bisulfite sequencing of the CpG island across the *TIM3* and *PD1* transcription start site (TSS). Open circles: Unmethylated CpG sites; Filled circles: Methylated CpG sites. (n = 2 replicates per group)

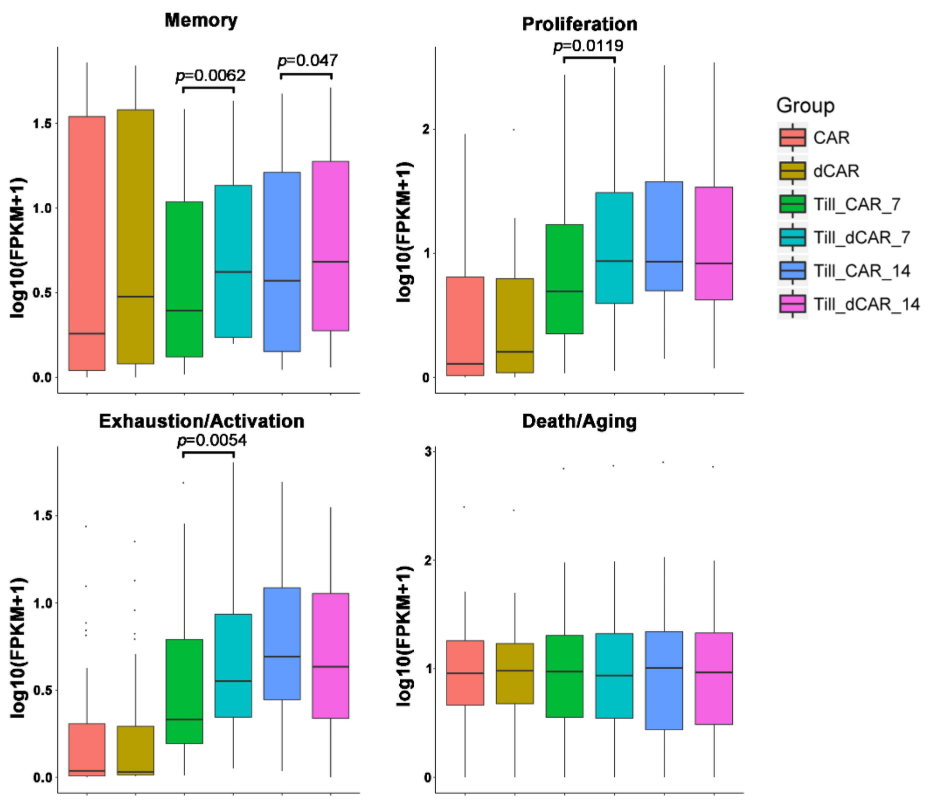

Supplementary Figure 10. Tumor-infiltrating dCAR T cells show increased effector and memory function compared to tumor-infiltrating CAR T cells. The box plots show the quartile of log-10 of the mean FPKM (fragments per kilobase of exon model per million mapped fragments) value from the appointed T cell function-associated genes. For the specified gene lists, see the source data. The expression of appointed T cell function-associated gene expression value in the tumour-infiltrating dCAR and tumour-infiltrating CAR T cells on days 7 and 14 after cell treatment. Mean average plots of genes differentially expressed in tumor-infiltrating dCAR T cells versus tumor-infiltrating CAR T cells at different time points after cell infusion. Box, median ± interquartile range; whiskers, 1.5× interquartile range. (n = 2). Pairwise comparisons were performed using a two-tailed, unpaired, t-tests.

Supplementary Table 1. Probe sequence of scFv-, FasL-, IFNγ- and a Granzyme B-specific probe

Probe name	Probe sequence
scFv	CTGCTCCACGCCGCCAGGCCGGAGGTGAAACTGCAGGAGTCAGGACCTGGCCTGG<br>TGGCGCCCTCACAGAGCCTGTCCGTCACATGCACTGTCTCAGGGGTCTCATTACCC<br>GACTATGGTGTAAGCTGGATTCGCCAGCCTCCACGAAAGGGTCTGGAGTGGCTGG<br>GAGTAATATGGGGTAGTGAAACCACATACTATAATTCAGCTCTCAAATCCAGACTG<br>ACCATCATCAAGGACAACTCCAAGAGCCAAGTTTTCTTAAAAATGAACAGTCTGCA<br>AACTGATGACACAGCCATTTACTACTGTGCCAAACATTATTACTACGGTGGTAGCT<br>ATGCTATGGACTACTGGGGTCAAGGAACCTCAGTCACCGTCTCCTCAGGTGGAGGC<br>GGTTCAGGCGGAGGTGGCTCTGGCGGTGGCGGATCGGACATCCAGATGACACAGA<br>CTACATCCTCCCTGTCTGCCTCTCTGGGAGACAGAGTCACCATCAGTTGCAGGGCA<br>AGTCAGGACATTAGTAAATATTTAAATTGGTATCAGCAGAAACCAGATGGAACTGT<br>TAAACTCCTGATCTACCATACATCAAGATTACACTCAGGAGTCCCATCAAGGTTCA<br>GTGGCAGTGGGTCTGGAACAGATTATTCTCTCACCATTAGCAACCTGGAGCAAGAA<br>GATATTGCCACTTACTTTTGCCAACAGGGTAATACGCTTCCGTACACGTTCG
FasL	CCAGCTGCCATGCAGCAGCCCTTCAATTACCCATATCCCCAGATCTACTGGGTGGA<br>CAGCAGTGCCAGCTCTCCCTGGGCCCCTCCAGGCACAGTTCTTCCCTGTCCAACCTC<br>TGTGCCCAGAAGGCCTGGTCAAAGGAGGCCACCACCACCACCGCCACCGCCACCA<br>CTACCACCTCCGCCGCCGCCGCCACCACTGCCTCCACTACCGCTGCCACCCCTGAA<br>GAAGAGAGGGAACCACAGCACAGGCCTGTGTCTCCTTGTGATGTTTTTCATGGTTC<br>TGGTTGCCTTGGTAGGATTGGGCCTGGGGATGTTTCAGCTCTTCCACCTACAGAAG<br>GAGCTGGCAGAACTCCGAGAGTCTACCAGCCAGATGCACACAGCATCATCTTTGG<br>AGAAGCAAATAGGCCACCCCAGTCCACCCCCTGAAAAAAAGGAGCTGAGGAAAGT<br>GGCCCATTTAACAGGCAAGTCCAACTCAAGGTCCATGCCTCTGGAATGGGAAGAC<br>ACCTATGGAATTGTCCTGCTTTCTGGAGTGAAGTATAAGAAGGGTGGCCTTGTGAT<br>CAATGAAACTGGGCTGTACTTTGTATATTCCAAAGTATACTTCCGGGGTCAATCTT<br>GCAACAACCTGCCCCTGAGCCACAAGGTCTACATGAGGAACTCTAAGTATCCCCAG<br>GATCTGGTGATGATGGAGGGGAAGATGATGAGCTACTGCACTACTGGGCAGATGT<br>GGGCCCGCAGCAGCTACCTGGGGGCAGTGTTCAATCTTACCAGTGCTGATCATTTA<br>TATGTCAACGTATCTGAGCTCTCTCTGGTCAATTTTGAGGAATCTCAGACGTTTTTC<br>GGCTTATATAAGCTCTAAGAGAAGCACTTTGGGATTCTTTCCATTATGATTCTTTGT<br>TACAGGCACCGAGAATGTTGTATTCAGTGAGGGTCTTCTTACATGCATTTGAGGTC<br>AAGTAAGAAGACATGAACCAAGTGGACCTTGAGACCACAGGGTTCAAAATGTCTG<br>TAGCTCCTCAACTCACCTAATGTTTATGAGCCAGACAAATGGAGGAATATGACGGA<br>AGAA CATAG
IFNγ	AAGAACTACTGATTTCAACTTCTTTGGCTTAATTCTCTCGGAAACGATGAAATATA<br>CAAGTTATATCTTGGCTTTTCAGCTCTGCATCGTTTTGGGTTCTCTTGGCTGTTACTG<br>CCAGGACCCATATGTAAAAGAAGCAGAAAACCTTAAGAAATATTTTAATGCAGGT<br>CATTCAGATGTAGCGGATAATGGAACTCTTTTCTTAGGCATTTTGAAGAATTGGAA<br>AGAGGAGAGTGACAGAAAAATAATGCAGAGCCAAATTGTCTCCTTTTACTTCAAA<br>CTTTTTAAAAACTTTAAAGATGACCAGAGCATCCAAAAGAGTGTGGAGACCATCA

	AGGAAGACATGAATGTCAAGTTTTTCAATAGCAACAAAAAGAAACGAGATGACTT<br>CGAAAAGCTGACTAATTATTCGGTAACTGACTTGAATGTCCAACGCAAAGCAATAC<br>ATGAACTCATCCAAGTGATGGCTGAACTGTCGCCAGCAGCTAAAACAGGGAAGCG<br>AAAAAGGAGTCAGATGCTGTTTCGAGGTCGAAGAGCATCCCAGTAATGGTTGTCCT<br>GCCTGCAATATTTGAATTTTAAATCTAAATCTATTTATTAATATTTAACATTATTTAT<br>ATGGGAATATATTTTTAGACTCATCAATCAAATAAGTATTTATAATAGCAACTTTT<br>GTGTAATGAAAATGAATATCTATTAATATATGTATTATTTATAATTCCTATATCCTG<br>TGACTGTCTCACTTAATCCTTTGTTTTCTGACTAATTAGGCAAGGCTATGTGATTAC<br>AAGGCTTTATCTCAGGGGCCAACTAGGCAGCCAACCTAAGCAAGATCCCATGGGTT<br>GTGTGTTTATTTCACTTGATGATACAATGAACACTTATAAGTGAAGTGATACTATCC<br>AGTTACTGCCGGTTTGAAAATATGCCTGCAATCTGAGCCAGTGCTTTAATGGCATG<br>TCAGACAGAACTTGAATGTGTCAGGTGACCCTGATGAAAACATAGCATCTCAGGA<br>GATTTCATGCCTGGTGCTTCCAAATATTGTTGACAACTGTGACTGTACCCAAATGG<br>AAAGTA
Granzyme B	AAGAGCTAAAAGAGAGCAAGGAGGAAACAACAGCAGCTCCAACCAGGGCAGCCT<br>TCCTAGAAGATGCAACCAATCCTGCTTCTGCTGGCCTTCCTCCTGCTGCCCAGGGC<br>AGATGCAGGGGAGATCATCGGGGGACATGAGGCCAAGCCCCACTCCCGCCCCTAC<br>ATGGCTTATCTTATGATCTGGGATCAGAAGTCTCTGAAGAGGTGCGGTGGCTTCCT<br>GATACGAGACGACTTCGTGCTGACAGCTGCTCACTGTTGGGGAAGCTCCATAAATG<br>TCACCTTGGGGGCCCACAATATCAAAGAACAGGAGCCGACCCAGCAGTTTATCCCT<br>GTGAAAAGACCCATCCCCCATCCAGCCTATAATCCTAAGAACTTCTCCAACGACAT<br>CATGCTACTGCAGCTGGAGAGAAAGGCCAAGCGGACCAGAGCTGTGCAGCCCCTC<br>AGGCTACCTAGCAACAAGGCCCAGGTGAAGCCAGGGCAGACATGCAGTGTGGCCG<br>GCTGGGGGCAGACGGCCCCCCTGGGAAAACACTCACACACACTACAAGAGGTGAA<br>GATGACAGTGCAGGAAGATCGAAAGTGCGAATCTGACTTACGCCATTATTACGAC<br>AGTACCATTGAGTTGTGCGTGGGGGACCCAGAGATTAAAAAGACTTCCTTTAAGGG<br>GGACTCTGGAGGCCCTCTTGTGTGTAACAAGGTGGCCCAGGGCATTGTCTCCTATG<br>GACGAAACAATGGCATGCCTCCACGAGCCTGCACCAAAGTCTCAAGCTTTGTACAC<br>TGGATAAAGAAAACCATGAAACGCTACTAACTACAGGAAGCAAACTAAGCCCCCG<br>CTGTAATGAAACACCTTCTCTGGAGCCAAGTCCAGATTTACACTGGGAGAGGTGCC<br>AGCAACTGAATAAATACCTCT
