# Supplementary material for: Low-dose decitabine priming endows CAR T cells with enhanced and persistent antitumour potential via epigenetic reprogramming
Source: Nat Commun. 2021 Jan 18;12:409. doi: 10.1038/s41467-020-20696-x (PMC7814040; doi:10.1038/s41467-020-20696-x)
Supplement: Supplementary file 3 — Descriptions of Additional Supplementary Files [file 41467_2020_20696_MOESM3_ESM.pdf]

Descriptions of Additional Supplementary Files

Supplementary Data 1

Description: Significant differentially methylation CpG sites among dCAR T cells compared to CAR T cells. Significantly differentially CpG sites were calculated by Generalized Linear Models (v3.36.2) (P value < 0.05, fold change (log2 scale) ≥ 1 or ≤ −1). (n=2)

Supplementary Data 2

Description: Significant differentially methylation CpG sites among dCAR T cells compared to CAR T cells after antigen stimulation. Significantly differentially CpG sites were calculated by Generalized Linear Models (v3.36.2) (P value < 0.05, fold change (log2 scale) ≥ 1 or ≤ −1). (n=2)

Supplementary Movie 1

Description: Antitumour reactivity of CAR T cells at low effector:target ratio. CAR T cells cocultured with Raji cells at an E:T ratio of 1:30. Green fluorescent (nuclearrestricted RFP) cells were CAR T cells. Red fluorescent (Yoyo3) cells were counted as dead cells. Phase and fluorescence (acquisition time, 400 ms) images were obtained every two hours for 64 hours in the IncuCyte S3 (37°C). Stacks of images were exported in tagged image file format (TIF) using the time plot function in the IncuCyte graph/export menu. Videos were assembled in Microsoft PowerPoint, exported in MP4 format.

Supplementary Movie 2

Description: Antitumour reactivity of dCAR T cells at low effector:target ratio. dCAR T cells cocultured with Raji cells at an E:T ratio of 1:30. Green fluorescent (nuclearrestricted RFP) cells were CAR T cells. Red fluorescent (Yoyo3) cells were counted as dead cells. Phase and fluorescence (acquisition time, 400 ms) images were obtained every two hours for 64 hours in the IncuCyte S3 (37°C). Stacks of images were exported in tagged image file format (TIF) using the time plot function in the IncuCyte graph/export menu. Videos were assembled in Microsoft PowerPoint, exported in MP4 format.
