# Supplementary material for: Low-dose decitabine priming endows CAR T cells with enhanced and persistent antitumour potential via epigenetic reprogramming
Source: Nat Commun. 2021 Jan 18;12:409. doi: 10.1038/s41467-020-20696-x (PMC7814040; doi:10.1038/s41467-020-20696-x)
Supplement: Supplementary file 8 — Reporting Summary [file 41467_2020_20696_MOESM8_ESM.pdf]

nature research

Corresponding author(s): Weidong Han

Last updated by author(s): 13/11/20

Reporting Summary

Nature Research wishes to improve the reproducibility of the work that we publish. This form provides structure for consistency and transparency in reporting. For further information on Nature Research policies, see our Editorial Policies and the Editorial Policy Checklist.

Statistics

For all statistical analyses, confirm that the following items are present in the figure legend, table legend, main text, or Methods section.

n/a	Confirmed	
☐	☒	The exact sample size (n) for each experimental group/condition, given as a discrete number and unit of measurement
☐	☒	A statement on whether measurements were taken from distinct samples or whether the same sample was measured repeatedly
☐	☒	The statistical test(s) used AND whether they are one- or two-sided<br>*Only common tests should be described solely by name; describe more complex techniques in the Methods section.*
☐	☒	A description of all covariates tested
☐	☒	A description of any assumptions or corrections, such as tests of normality and adjustment for multiple comparisons
☐	☒	A full description of the statistical parameters including central tendency (e.g. means) or other basic estimates (e.g. regression coefficient) AND variation (e.g. standard deviation) or associated estimates of uncertainty (e.g. confidence intervals)
☐	☒	For null hypothesis testing, the test statistic (e.g. F, t, r) with confidence intervals, effect sizes, degrees of freedom and P value noted<br>*Give P values as exact values whenever suitable.*
☒	☐	For Bayesian analysis, information on the choice of priors and Markov chain Monte Carlo settings
☒	☐	For hierarchical and complex designs, identification of the appropriate level for tests and full reporting of outcomes
☒	☐	Estimates of effect sizes (e.g. Cohen's d, Pearson's r), indicating how they were calculated

Our web collection on statistics for biologists contains articles on many of the points above.

Software and code

Policy information about availability of computer code

Data collection	Flow cytometry data were collected using Cytexpert software (version 1.1.10.0, BECKMAN COULTER). Cell lysis was measured by Varioskan™ LUX (Thermo Fisher). Cytotoxicity assays were monitored using an xCELLigence Real-Time Cell Analyzer-Multiple Plate system (Agilent Technologies). Bioluminescence imaging for animal studies was collected using NightOwl Ⅱ platform (LB 983, Berthold). Q-PCR data were collected using ABI prism 7500 sequence detection system (Applied Biosystems).The purity and concentration of DNA was estimated using Nanodrop 2000 (ThermoScietific). Genome-wide DNA methylation was assessed using the Illumina Infinium HumanMethylation850K BeadChip (Illumina Inc, USA).
Data analysis	FACS data were analyzed by Cytexpert software (version 1.1.10.0, BECKMAN COULTER) and FlowJo software (Version 10.0.7, FlowJo, Ashland, OR). Western blot data were analyzed by ImageJ software (Version 1.46r, National Institutes of Health). Bioluminescence images from animal studies were analyzed using indiGO software (Berthold). RNA sequencing data were analyzed using the FastQC v0.11.3, Hisat2 v2.0.5, edgeR package, DEseq2 package version 1.16.1, topHat and cufflinks. Statistical analyses were performed using Microsoft Excel version 2010 or GraphPad Prism 7 software (GraphPad Software Inc.). The array data (.IDAT files) was analysed using package in R software v3.5. for deriving the methylation level.

For manuscripts utilizing custom algorithms or software that are central to the research but not yet described in published literature, software must be made available to editors and reviewers. We strongly encourage code deposition in a community repository (e.g. GitHub). See the Nature Research guidelines for submitting code & software for further information.

Data

Policy information about availability of data

All manuscripts must include a data availability statement. This statement should provide the following information, where applicable:

- Accession codes, unique identifiers, or web links for publicly available datasets
- A list of figures that have associated raw data
- A description of any restrictions on data availability

All relevant data related to this manuscript are available on request from the authors on reasonable request. The accession number for the RNA-sequencing data described in this study is GSE156207. The accession number for the Methylation-sequencing data described in this study is GSE161506. Original un-cropped western blots are provided in source data. The source data underlying Figs. 1–10 and Supplementary Figs. 1–10 are provided.

Field-specific reporting

Please select the one below that is the best fit for your research. If you are not sure, read the appropriate sections before making your selection.

☒ Life sciences ☐ Behavioural & social sciences ☐ Ecological, evolutionary & environmental sciences

For a reference copy of the document with all sections, see nature.com/documents/nr-reporting-summary-flat.pdf

Life sciences study design

All studies must disclose on these points even when the disclosure is negative.

Sample size	A statistical method was not used to determine sample size. For in vitro studies, a minimum of triplicates was chosen to allow for calculation of statistics. For animal studies, 5-10 mice were used in full-scale studies based on our prior experience with the Raji and NALM6 tumor model indicating this sample size would likely enable detection of statistically significant differences in survival across different CAR designs based on log-rank analysis across test groups.<br>All experiments were performed multiple times and conclusions were considered valid if results were reproducible between experiments.
Data exclusions	No data was excluded from the analysis.
Replication	All major experiments were repeated with similar results as stated in the figure legends.
Randomization	For in vitro experiments, samples were randomly allocated into different groups. In animal studies, mice were assigned randomly to experimental and control groups. Tumor burden was determined by bioluminescent imaging one day prior to CAR T cell transfer. Since tumor burdens are very even with the NALM6 or Raji cell lines, no mice were excluded prior to treatment and mice were randomly assigned into treatment groups.
Blinding	Mouse condition and survival were observed by an operator who was blinded to treatment groups in addition to a blinded operator was measured Tumor burden. Group allocation and analysis of data were not performed in blinded fashion due to the need to make decisions on next-steps in the project based on emerging in vivo results, before long-term studies fully concluded. All data analyses are based on objectively measurable data (fluorescence intensity, tumor burden, cell count, gene expression level). For other experiments, care was taken to make sure that all samples were uniformly processed and analyzed to ensure consistency between control and test samples to prevent study bias. Since the results reported are primarily quantitative (e. not subjective evaluation of behavioral changes, etc. h blinding was not necessary for the experiments.

Reporting for specific materials, systems and methods

We require information from authors about some types of materials, experimental systems and methods used in many studies. Here, indicate whether each material, system or method listed is relevant to your study. If you are not sure if a list item applies to your research, read the appropriate section before selecting a response.

Materials & experimental systems

n/a	Involved in the study	
☐	☒	Antibodies
☐	☒	Eukaryotic cell lines
☒	☐	Palaeontology and archaeology
☐	☒	Animals and other organisms
☐	☒	Human research participants
☒	☐	Clinical data
☒	☐	Dual use research of concern

Methods

n/a	Involved in the study	
☒	☐	ChIP-seq
☐	☒	Flow cytometry
☒	☐	MRI-based neuroimaging

Antibodies

Antibodies used

Antibody name	Clone	Cat.No.	Vendor
APC-CD3	UCHT1	555335	Becton,Dickinson and Company
PerCP-CD3	SP34-2	552851	Becton,Dickinson and Company
PE-CD4	L200	550630	Becton,Dickinson and Company
FITC-CD4	L200	550628	Becton,Dickinson and Company
PE-CD8	RPA-T8	555367	Becton,Dickinson and Company
FITC-CD8	561947	555367	Becton,Dickinson and Company
APC-cy7-CD8	RPA-T8	557760	Becton,Dickinson and Company
APC-CD62L	DREG-56	559772	Becton,Dickinson and Company
FITC-CD45RO	UCHL1	555492	Becton,Dickinson and Company
APC-CD45RO	UCHL1	560899	Becton,Dickinson and Company
PE-PD1	EH12.1	560795	Becton,Dickinson and Company
APC-PD1	MIH4	558694	Becton,Dickinson and Company
APC-CD25	M-A251	561399	Becton,Dickinson and Company
BV510-LAG3	T47-530	744985	Becton,Dickinson and Company
PE-Foxp3	259D/C7	562421	Becton,Dickinson and Company
IL-17A	N49-653	560486	Becton,Dickinson and Company
APC-CD107a	H4A3 5	560664	Becton,Dickinson and Company
FITC-EOMES	WD1928	11-4877-42	eBioscience
FITC-TIM3	RUO	345021	Biolegend
APC-TIM3	F38-2E2	345012	Biolegend
APC-Cy7-HLA-DR	L243	307618	Biolegend
PE-KI67	REA183	130120417	Miltenyi Biotec
PE-streptavidin		554062	Becton,Dickinson and Company
FITC-streptavidin		554060	Becton,Dickinson and Company
anti-beta-actin	mAbcam8226	ab8226	Abcam
anti-Dnmt3a	EPR18455	ab188470	Abcam

Biotin-SP-AffiniPure F(ab)'2 fragment-specific goat anti-mouse IgG antibody 115-066-072 (Jackson ImmunoResearch)
FITC-conjugated AffiniPure F(ab)'2 fragment-specific goat anti-mouse IgG antibody 115-095-072 (Jackson ImmunoResearch)

Validation

Antibody identity was used as indicated by the manufacturers, which are identified in the Methods section. The manufacturers rovided certificates of analysis; no additional validation was performed in house. For flow cytometry experiments, titration was rformed for each antibody to determine the appropriate staining concentration. Positive and negative samples were stained at varying dilutions. In the case where negative samples were not available, corresponding isotype control antibodies were used The staining concentration used had the largest dynamic range between positive and negative samples.

Eukaryotic cell lines

Policy information about cell lines

Cell line source(s)

The Burkitt lymphoma cell line Raji, B lymphocyte leukemia cell line Nalm-6 and chronic myelogenous leukaemia cell line K562 were purchased from ATCC (Manassas, VA); 293T cell was purchased from Cell Bank of Chinese Academy of Medical Sciences.

Authentication

Cell phenotypes were assessed by flow cytometry.

Mycoplasma contamination

All cell lines were routinely tested or mycoplasma and were found to be negative.

Commonly misidentified lines
(See ICLAC register)

No commonly misidentified cell lines were used.

Animals and other organisms

Policy information about studies involving animals; ARRIVE guidelines recommended for reporting animal research

Laboratory animals

6-8 week-old female NOD-Prkdcscid-Il2rgnull/Vst (NPG) mice (Beijing Vitalstar Biotechnology).

Wild animals

Wild animals were not used.

Field-collected samples

Field-collected samples were not used.

Ethics oversight

All mouse experiments were approved by the Chinese People's Liberation Army General Hospital Laboratory Animal Center, China.

Note that full information on the approval of the study protocol must also be provided in the manuscript.

Human research participants

Policy information about studies involving human research participants

Population characteristics	PBMCS from anonymous healthy donors from the Chinese People's Liberation Army general hospital. The researchers were blind to any covariate characteristics.
Recruitment	There was no recruitment of participants PBMCS were get from the Chinese People's Liberation Army generalhospital as described.
Ethics oversight	The study was undertaken in accordance with the approval of the Chinese People's Liberation Army General Hospital Ethics Committee, China.

Note that full information on the approval of the study protocol must also be provided in the manuscript.

Flow Cytometry

Plots

Confirm that:

- [x] The axis labels state the marker and fluorochrome used (e.g. CD4-FITC).
- [x] The axis scales are clearly visible. Include numbers along axes only for bottom left plot of group (a 'group' is an analysis of identical markers).
- [x] All plots are contour plots with outliers or pseudocolor plots.
- [x] A numerical value for number of cells or percentage (with statistics) is provided.

Methodology

Sample preparation	Samples from in vitro studies included primary T cells as well as immortalized cell lines. Samples from animal studies were collected from mice, tumors were cut into fine pieces, filtered through a 100 um cell strainer and washed with PBS prior to antibody staining. Prior to flow cytometry, samples were washed in PBS and stained with antibodies. For analysis of peripheral blood and bone marrow, plasma was removed by low speed centrifugation, cells were resuspended in PBS, and whole blood was stained with antibodies prior to fixation of cells and lysis of RBCs.
Instrument	Cytexpert software (version 1.1.10.0, BECKMAN COULTER)
Software	FACS data were collected and analyzed by Cytexpert software (version 1.1.10.0, BECKMAN COULTER) and FlowJo software (Version 10.0.7, FlowJo, Ashland, OR).
Cell population abundance	The purity was verified by flow cytometry.
Gating strategy	FSC-A/FSC-H plots were used to determine singlet ates FSC-A/SSC-A plots were used to determine cell population gates otype controls were used to indicate the boundaries between positive and negative populations.

- [x] Tick this box to confirm that a figure exemplifying the gating strategy is provided in the Supplementary Information.
